# Supplementary material for: Effect of hearing aids on body balance function in non-reverberant condition: A posturographic study
Source: PLoS One. 2021 Oct 13;16(10):e0258590. doi: 10.1371/journal.pone.0258590 (PMC8513876; doi:10.1371/journal.pone.0258590)
Supplement: Attachment — Submitted filename: Response to Reviewers.docx [file pone.0258590.s001.docx]

Dear Professor Alimohamad Asghari and Professor Salwa Mahmoud,

Thank you for positive and constructive comments. We revised the paper according to your recommendation.

Reviewer #1: The study is interesting but it has some limitations such as small number of participants that authors mentioned.

**1) All of the normal hearing cases were female. why did the authors choose just females for normal hearing group?**

**Answer.**

Thank you for the suggestion. We recruited normal participants regardless of the gender. Most of the attendee were unexpectedly female. In addition, all the attended males did not fulfil our inclusion criteria (mainly high pure tone threshold).

The following descriptions were added.

L288

“Second, the normal participants in the present study were all female. We recruited normal-hearing paid volunteers regardless of the gender. Most of the attendee were female, and none of the male attendees met the inclusion criteria. This may be due to the high prevalence of age-related hearing loss in men (18). Recent posturographic study showed that the gender difference was statistically significant (19). Despite that, females are known to be more prone to falls than males. It may be preferable to recruit gender-matched control participants in further studies.”

**2) In table 2, it is better to add "p values" in the table.**

**Answer.**

We made the table according to the previous report (ref #5, Negahban et al., 2017). As the reviewer #1 suggested, it is better to include the result of statistics in the table. The problem is that we used three-way repeated measures and mixed factorial ANOVA. We like this procedure, but it is not easy to include the results of ANOVA in the table.

The following is a reformatted table that include the results of ANOVA. This table is informative but I feel it too busy. I like the original table, but am ready to use the reformatted table if the reviewer like the new one.

|  |  |  | HA users | | NH participants | |
| --- | --- | --- | --- | --- | --- | --- |
|  |  |  | Mean (SEM) | | Mean (SEM) | |
|  |  |  | Sound(+) | Sound(-) | Sound(+) | Sound(-) |
| Total Area  Significant interaction between the sound condition and foam condition (F [1, 18] =7.04, *p* = 0.02), between sound condition and eye condition (F [1, 18] = 5.77, *p* = 0.03), and between eye and foam conditions (F [1, 18] = 96.66, *p* < 0.01) | | | | | | |
|  | Eyes open-Rigid surface |  | 3.04(0.87) | 2.98(0.96) | 2.21(0.30) | 1.97(0.27) |
|  | Eyes open-Foam surface |  | 5.41(1.44) | 5.76(1.35) | 4.79(0.66) | 5.56(0.63) |
|  | Eyes closed-Rigid surface |  | 5.48(1.04) | 6.11(1.20) | 4.09(0.69) | 4.52(0.43) |
|  | Eyes closed-Foam surface |  | 25.47(3.73) | 28.94(3.58) | 18.66(1.65) | 21.90(1.61) |
| Maximum displacement, ML  Significant interaction among the eye condition, sound condition, and between-group factor (F [1, 18] = 6.19, *p* = 0.02) | | | | | | |
|  | Eyes open-Rigid surface |  | 2.25(0.33) | 2.31(0.32) | 1.98(0.18) | 1.88(0.17) |
|  | Eyes open-Foam surface |  | 3.06(0.39) | 3.24(0.50) | 2.78(0.16) | 3.27(0.19) |
|  | Eyes closed-Rigid surface |  | 3.62(0.36) | 3.59(0.42) | 2.80(0.29) | 3.40(0.20) |
|  | Eyes closed-Foam surface |  | 7.83(0.49) | 8.12(0.60) | 6.54(0.41) | 7.48(0.33) |
| Maximum displacement, AP  Significant main effect of sound condition (F (1, 18) = 7.95, *p* = 0.01) and a significant interaction between eye condition and foam condition (F (1, 18) =70.88, *p* < 0.01) | | | | | | |
|  | Eyes open-Rigid surface |  | 2.80(0.40) | 2.79(0.52) | 2.65(0.23) | 2.41(0.16) |
|  | Eyes open-Foam surface |  | 3.76(0.40) | 3.78(0.31) | 3.69(0.36) | 4.34(0.34) |
|  | Eyes closed-Rigid surface |  | 3.46(0.35) | 3.66(0.37) | 3.21(0.32) | 3.32(0.22) |
|  | Eyes closed-Foam surface |  | 7.01(0.50) | 7.88(0.58) | 6.83(0.33) | 6.99(0.54) |
| Sway Velocity  Significant main effect of sound condition (F (1, 18) = 9.06, *p* < 0.01) and a significant interaction between the eye condition and foam condition (F (1, 18) =74.75, *p* < 0.01) | | | | | | |
|  | Eyes open-Rigid surface |  | 1.48(0.23) | 1.73(0.30) | 1.11(0.07) | 1.11(0.08) |
|  | Eyes open-Foam surface |  | 2.29(0.35) | 2.35(0.25) | 2.08(0.11) | 2.21(0.15) |
|  | Eyes closed-Rigid surface |  | 2.43(0.24) | 2.55(0.20) | 1.80(0.16) | 2.01(0.19) |
|  | Eyes closed-Foam surface |  | 6.52(0.80) | 6.93(0.80) | 5.10(0.55) | 5.27(0.30) |
| Velocity, ML  Significant main effect of sound condition (F (1, 18) = 6.53, *p* = 0.02) and a significant interaction between the eye condition and foam condition (F (1, 18) =66.08, *p* < 0.01) | | | | | | |
|  | Eyes open-Rigid surface |  | 0.80(0.11) | 0.94(0.14) | 0.62(0.05) | 0.64(0.05) |
|  | Eyes open-Foam surface |  | 1.21(0.17) | 1.27(0.14) | 1.12(0.05) | 1.18(0.06) |
|  | Eyes closed-Rigid surface |  | 1.35(0.14) | 1.43(0.15) | 1.00(0.09) | 1.23(0.12) |
|  | Eyes closed-Foam surface |  | 3.32(0.42) | 3.61(0.43) | 2.91(0.33) | 2.92(0.14) |
| Velocity, AP  Significant main effect of sound condition (F (1, 18) = 10.44, P < 0.01) and a significant interaction between the eye condition and foam condition (F (1, 18) =94.10, P < 0.01) | | | | | | |
|  | Eyes open-Rigid surface |  | 0.80(0.15) | 0.94(0.20) | 0.62(0.05) | 0.61(0.05) |
|  | Eyes open-Foam surface |  | 1.22(0.20) | 1.25(0.08) | 1.19(0.07) | 1.28(0.11) |
|  | Eyes closed-Rigid surface |  | 1.31(0.16) | 1.36(0.10) | 1.02(0.11) | 1.01(0.10) |
|  | Eyes closed-Foam surface |  | 3.51(0.38) | 3.66(0.37) | 2.80(0.18) | 2.95(.019) |

**3) Although the authors found significant difference in posturography values but they did not evaluate the balance status by other tests to show its "clinical importance".**

**Answer.**

Thank you for the advice.

I agree with the referee that it is better to include participants with vestibulopathy.

We tested the participants with vestibulo-ocular reflex test and head impulse test to exclude severe vestibulopathy, as was conducted in the previous study (ref #4, Vitkovic et al., 2016). However, we found that participants with vestibulopathy cannot complete the experiment with foam surface (ref #9, Oikawa et al., 2018). Based on this finding, we conducted the present study only in those with normal vestibular functions.

The following descriptions were added.

L79

“None of them showed abnormalities in vestibular test including vestibulo-ocular reflex test and head impulse test.”

L294

“All the participants were of normal vestibular function. Further studies exploring dynamic and reverberant conditions and vestibulopathy participants are warranted.”

**4) The figures are difficult to interpret.**

**Answer.**

In this study, we used three-way repeated measures and mixed factorial ANOVA. The figures were made to represent the results of the statistics. The figures can be made simple if we use the average and SEM, but it will reduce the individual information. We made the figures so as not to delete the raw data. In addition, we can compare the velocity and displacement in hearing aid users with eyes closed (Fig 1(a) and left column of Fig 2).

I will appreciate it if the reviewers accept the figures in the present form.

Reviewer #2:

**1) Please give more details for auditory distance perception**

**Answer.**

Thank you for the advice. We add more detail about the auditory distance perception.

L286

“Second, the normal participants in the present study were all female. We recruited normal-hearing paid volunteers regardless of the gender. Most of the attendee were female, and none of the male attendees met the inclusion criteria. This may be due to the high prevalence of age-related hearing loss in men (18). Recent posturographic study showed that the gender difference was not statistically significant (19). Despite that, females are known to be more prone to falls than males. It may be preferable to recruit gender-matched control participants in further studies.”

**2) Make sure that no other dual publication for this paper not occurred**

**Answer.**

I confirm that no other dual publication for this paper occurred.

**3) You must follow the research ethical rules for privacy and honesty for patients**

**Answer.**

This study was conducted in accordance with the Declaration of Helsinki. All the identifying information were removed from the analyzed data.
